# Advancing and translating knowledge: a systematic inquiry into the 2010–2020 mental health and psychosocial support intervention research evidence base

**DOI:** 10.1017/gmh.2022.6

**Published:** 2022-03-23

**Authors:** Ashley Nemiro, Theresa Jones, Olivia Tulloch, Leslie Snider

**Affiliations:** 1 The MHPSS Collaborative, Save the Children Denmark Rosenørns Allé 12, 1634 Copenhagen V, Denmark; 2 Anthrologica, Woad Mill, Broughton, Oxfordshire, OX15 6AR, UK

**Keywords:** Mental health and psychosocial support (MHPSS), intervention research, humanitarian settings, evidence review

## Abstract

**Background and study objectives:**

MHPSS is increasingly seen as a critical component to effective and responsible humanitarian programming. This review examines the extent to which MHPSS research generated since 2010 has contributed to the public health evidence base and how this has influenced and impacted programming and policy in humanitarian settings.

**Methods:**

This mixed-method study included a scoping literature review (*n* = 50) and a consultation process with qualitative key informant interviews (*n* = 19) and online survey responses (*n* = 52) to identify the facilitating and inhibiting factors for the two areas of inquiry and to understand the broader context in which knowledge is generated and taken up. The interviews were thematically analysed and the survey responses were descriptively analysed.

**Results:**

The review identified a rapidly growing evidence base that has evaluated a range of MHPSS interventions. However, few studies examined long-term impacts of interventions, there was limited direct evidence on outcomes for children and adolescents and whole family approaches, and there were minimal replications of the same approach that could test efficacy across settings and population groups. A general shift was identified in the consultation process away from a focus on disorder towards the more positive aspects of wellbeing. However, there remained a mismatch in many studies included in the literature review, whereby the interventions were broad, community-based but the outcome measures used still focused on changes in symptoms of mental disorders.

**Conclusion:**

The evidence base for MHPSS has grown significantly over the last 10 years. However, several knowledge gaps remain, as does the divide between research and practice. Moving forward, MHPSS intervention research needs to be more responsive to the needs on the ground.

## Background

The number of people forcibly displaced by conflict, violence and persecution reached a record high in 2020, estimated at 82.4 million (United Nations High Commissioner for Refugees, 2021). The mental health and psychosocial wellbeing of refugees, displaced persons and those affected by conflict or natural disaster is negatively impacted. It is estimated that more than one in five people in post-conflict settings have depression, anxiety disorder, post-traumatic stress disorder (PTSD), bipolar disorder or schizophrenia, and that almost one in 10 people have a moderate or severe mental disorder (Charlson *et al*., 2019). Although the prevalence of mental disorders and psychological distress is high and has known burdens, the expenditure is low (World Health Organization, 2017), and access to quality care in conflict settings is grossly inadequate. In 2005, the Inter-Agency Standing Committee (IASC) formed a task force that developed the Guidelines on Mental Health and Psychosocial Support in Emergency Settings (IASC, 2007) which was a catalyst for addressing the wellbeing of communities affected by humanitarian crises.

MHPSS is increasingly seen as a critical, cross-sectoral component to effective and responsible humanitarian programming (Meyer and Morand, 2015) and continues to gain recognition as a core component of any response. However, MHPSS research has not always reflected what is happening in practice in humanitarian settings. Historically, MHPSS research has largely concentrated on identifying the rates of PTSD and depression (Moore *et al*., 2020). Non-specific forms of psychological distress and psychosocial problems have been less well-researched, despite being the target of most MHPSS programmes in emergencies. A number of systematic reviews have been conducted in recent years on MHPSS intervention research; these have tended to limit their scope to interventions targeting mental health ‘disorders’ (e.g. McPherson, 2012; Meffert and Ekblad, 2013; Purgato *et al*., 2018*a*, 2018*b*; Thompson *et al*., 2018; Yohannan and Carlson, 2019) and/or to controlled or randomised controlled trials at the exclusion of other research designs (e.g. Brown *et al*., 2017; Purgato *et al*., 2018*b*; Bangpan *et al*., 2019). Reviews with a wider scope (e.g. Gouweloos *et al*., 2014; Pedersen *et al*., 2015; Jordans *et al*., 2016; Bangpan *et al*., 2019; Dickson and Bangpan, 2018; Haroz *et al*., 2020) have not combined their findings with an analysis of how this research has been taken up in policy and practice.

Tol *et al*. (2011*a*) concluded that little of the research focused on MHPSS interventions generated between 2000 and 2010 had been translated to practice and there was an urgent need to understand approaches that were commonly used in practice and that target the broad MHPSS needs of those affected by humanitarian crises. This would require close collaboration between researchers and practitioners, attention to sociocultural context, and amplifying the voice of those directly affected by humanitarian crises. Tol *et al*. (2011*b*) subsequently defined a consensus-based MHPSS research agenda including 10 priority research questions to guide the field and advance research to inform practice.

The review presented here was subsequently commissioned by Elrha to examine the extent to which MHPSS research generated since 2010 has contributed to the public health evidence base and how this has influenced and impacted programming and policy in humanitarian settings. In this paper, we present findings from two areas of inquiry from this review: how knowledge on MHPSS interventions researched in humanitarian settings has advanced in the past 10 years and how the evidence has been adopted into policy and practice at various levels of the humanitarian system.

## Methods

The review used mixed methods to identify the facilitating and inhibiting factors for the two areas of inquiry and to understand the broader context in which knowledge is generated and taken up.

### Literature review

The team conducted a scoping literature review informed by the review questions in Table 1.

**Table 1.** Review questions

Review questions What range of MHPSS interventions does the body of evidence cover? What is the geographical coverage of the evidence? What populations have been included? What is the nature of the research designs used? What types of outcome measures are used? To what extent does this body of evidence match the MHPSS research priorities set by Tol *et al*. in 2010? What is the quality of the evidence? To what extent has this evidence informed global MHPSS guidelines and strategy? What further gaps in research have been identified by the studies?

Selection criteria included studies published between January 2010 and April 2020 that described the testing, trialling or evaluation of MHPSS interventions delivered in humanitarian settings that were *not* focused on mental disorders (e.g. PTSD, depression). As noted in the introduction, in reality most MHPSS programmes do not target people with a diagnosable mental disorder. We wanted to understand the scope and range of research on MHPSS interventions that do not set out to treat mental disorders – e.g., those interventions that are used more in practice.

Studies were included on interventions that targeted non-specific psychological distress or wellbeing and other related psychosocial outcomes as their primary outcome measures (or, for qualitative studies, as their primary focus). In addition, studies were included on interventions that were explicit in not targeting people meeting criteria for a mental disorder, even if scales for mental disorders were included as outcome measures, as long as the study was not looking for clinical change on these measures.

Interventions included those integrated into basic humanitarian service provision, activities focused on community and family support, and psychological or social activities, provided in order to achieve MHPSS-related outcomes. Studies had to be based in lower-income countries or lower-middle income countries, in the context of a humanitarian response by governmental or non-governmental organisations (NGOs) to address the immediate impacts, aftermath or consequences of an emergency, including war, conflict, natural disaster and epidemic. The MHPSS interventions identified in the study targeted adults, children and/or adolescents. Inclusion and exclusion criteria are shown in Table 2.

**Table 2.** Inclusion and exclusion criteria

Inclusion criteria Studies published since 2010 that described the testing, trialling or evaluation of MHPSS interventions delivered in humanitarian settings that were not focused on mental disorders (e.g. PTSD, depression). Relevant MHPSS interventions included those integrated into basic humanitarian service provision, activities focused on community and family support, and psychological or social activities considered to be ‘interventions’, provided in order to achieve MHPSS-related outcomes. Both stand-alone interventions and those integrated into other sectors of a response, such as MHPSS and nutrition; MHPSS and disaster prevention; MHPSS and gender-based violence (GBV) prevention, etc. Studies based in the context of a humanitarian response, where governmental or non-governmental organisations (NGOs) were supporting a country to deal with the immediate impacts, the aftermath or the consequences of an emergency, including war, conflict, natural disaster and epidemics. Studies included those targeting both adults and children. Studies of both the outcomes of interventions and studies with a focus on participant experiences and perspectives (including from process evaluations). Where a purely quantitative design was used to measure outcomes, including experimental, quasi-experimental and prospective cohort studies, only controlled designs were included.
Exclusion criteria Studies published before 2010. Non-English language studies. Studies from non-humanitarian/crisis-affected settings, including HIV and poverty contexts. Studies from high-income countries (HICs) and upper middle-income countries (UMICs) as defined by www.data.worldbank.org. Systematic reviews. Multiple analyses of the same data pool (the most comprehensive study was retained). Studies of interventions with no mental health or psychosocial wellbeing target/outcome specified from the outset. Studies focusing on PTSD and/or other mental disorders with interventions that stated their primary orientation as being to treat clinical disorders. Studies in which primary outcome measures were disorder-focused and clinical change was expected through delivery of the intervention.

Intervention outcome studies and studies focusing on participant experiences (including from process evaluations) were included. Where a purely quantitative design was used to measure outcomes, including experimental, quasi-experimental and prospective cohort studies, only controlled designs were included. Comparison groups could be those with no intervention, on a waiting list, other active interventions or usual care. Qualitative and mixed-methods studies were included even where the quantitative component had no pre-test or control group.

The following international databases were searched: PsycINFO (peer-reviewed), Medline, Web of Science, the Cochrane Library and Google Scholar. The search terms, as set out in Table 3, were adapted as appropriate to the syntax of each database (see Table 4 for example search strategy and tables of search terms; Nemiro *et al*., 2021). When searching academic literature, the bibliographies of relevant studies were reviewed through ancestor searching and snowballing of sources. The reference lists of recent, relevant systematic reviews that included literature published since 2010 were hand-searched.

**Table 3.** Search terms

Search concept	Search terms (used alone or in combination)
Setting	Humanitarian; Crisis; War; Conflict; Emergency; Epidemic; Genocide; Earthquake; Flood; Famine; Drought; Tsunami; Terror; Trauma; Violence; Accident; Refugee; Migrant; Displaced; Disaster
Outcomes	Wellbeing; Well-being; Mental Health; Stress reduction; Functioning; Hope; Self-efficacy; Resilience; Reconciliation; Social connectedness; Social cohesion; Coping; Distress; Social support
Intervention	Psychosocial support; Psychosocial intervention; Psychological support; Psychological intervention; Mental Health support; Mental Health intervention; Social support; MHPSS; Psychotherapeutic; Counselling; Socio-therapy; Support groups; Peer support; Community healing dialogues; Communal healing; Psychoeducation; Community support; Family support; Social networks; Self-care; Self care; Self-help; Self help; Safe Space; Child Friendly Space; Psychological First Aid; Psychosocial Considerations
Study type	Intervention; trial; program; pilot

**Table 4.** Example search strategy

Web of Science core collection	
(1) Setting	TS = (Humanitarian OR Crisis OR War OR Conflict OR Emergency OR Epidemic OR Genocide OR Earthquake OR Flood OR Famine OR Drought OR Tsunami OR Terror OR Trauma OR Violence OR Accident OR Refugee OR Migrant OR Displaced OR Disaster)
(2) Outcomes	TS = (Wellbeing OR ‘Well-being’ OR ‘Mental Health’ OR ‘Stress reduction’ OR Functioning OR Hope OR ‘Self-efficacy’ OR Resilience OR Reconciliation OR ‘Social connectedness’ OR ‘Social cohesion’ OR coping OR distress OR ‘social support’)
(3) Interventions	TS = (‘Psychosocial support’ OR ‘Psychosocial intervention’ OR ‘Psychological support’ OR ‘Psychological intervention’ OR ‘Mental Health support’ OR ‘Mental Health intervention’ OR ‘Social support’ OR ‘MHPSS’ OR ‘Psychotherapeutic’ OR ‘Counselling’ OR ‘Socio-therapy’ OR ‘Support group’ OR ‘Peer support’ OR ‘Community healing dialogue’ OR ‘Communal healing’ OR ‘Psychoeducation’ OR ‘Community support’ OR ‘Family support’ OR ‘Social network’ OR ‘Self-care’ OR ‘Self care’ or ‘Self-help’ OR ‘self help’ OR ‘Safe Space*’ OR ‘Child Friendly Space’ OR ‘Psychological First Aid’ OR ‘psychosocial consideration’)
(4) Study type	TS = (Intervention OR trial OR program OR pilot)
1 and 2 and 3 and 4	
	Limitations: 2010–2020; English

TS includes title, abstract and key words.

The grey literature search included the resources or publications section of the websites of 27 organisations and agencies working on MHPSS in emergencies (Nemiro *et al*., 2021), the Intervention Journal website and four other specialist research websites (socialprotection.org, MHPSS.net, mhinnovation.net and the Regional Psychosocial Support Initiative). The resource pages of all websites were searched for ‘mental health’, ‘psychological’, ‘psychosocial’.

Sources were imported into a citation management software to facilitate inventory, cross-checking, removal of duplications and for further screening. The review team screened titles and abstracts against the inclusion criteria, to identify potentially eligible studies for which the full paper was retrieved. One reviewer screened titles and abstracts to select full papers and a second reviewer checked the abstract of papers that did not meet the inclusion criteria and reviewed the full paper if it was not clear from the abstract. Full papers were screened and identified for inclusion by one reviewer, and a second reviewer independently screened 25% of papers. Any discrepancies were discussed and resolved.

**Fig. 1.**
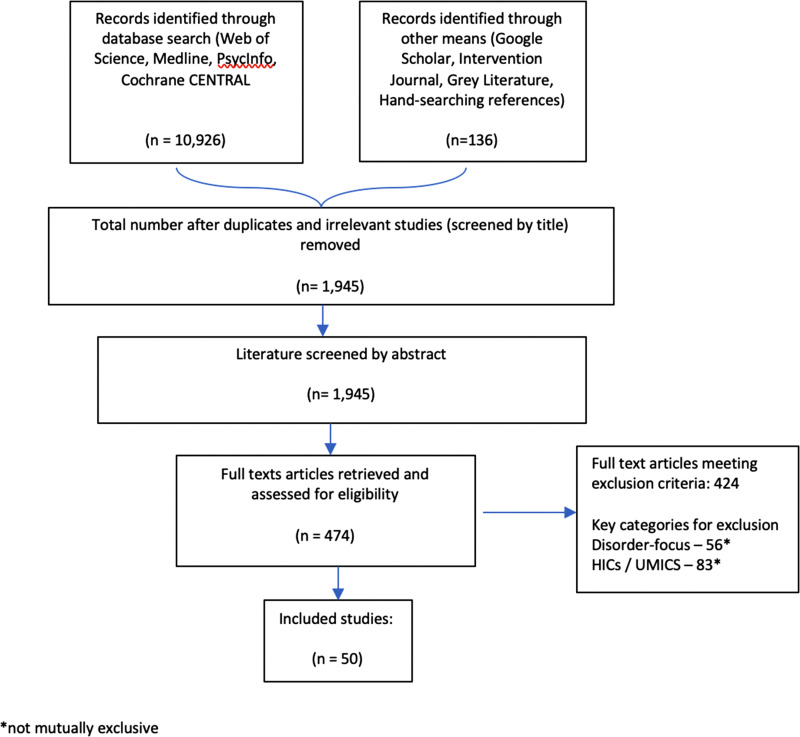
Flow of studies.

A data extraction matrix was developed based on the review questions in Fig. 1 and was piloted using a random sample of five articles and revised accordingly. The data of all pre-selected full-text sources were extracted. Data were synthesised into summary data tables to help illustrate the results of individual studies. A narrative synthesis of the findings was structured according to the study aims and review questions.

The review aimed to capture a broad landscape of existing evidence, which meant including studies that may be excluded from formal systematic literature reviews. Nevertheless, a quality appraisal process was applied to all selected studies. The rigour of studies exploring participants experiences and perspectives using qualitative methods was assessed according to the methodological criterion used by Bangpan *et al*. (2017) who re-purposed EEPI-Centre tools (Hurley *et al*., 2013; Brunton *et al*., 2016; Hurley *et al*., 2018) to assess the quality of study designs in a large-scale MHPSS systematic review process. Risk of bias was assessed in the RCTs and controlled before-and-after studies, using the criteria outlined in the Cochrane Handbook for Systematic Reviews of Interventions (Higgins *et al*., 2011). For the studies using a cohort design, an adaptation of the Newcastle-Ottowa Scale (Wells *et al*., 2019) was used to review quality.

### Consultation

The consultation process included key informant interviews and an online survey. It aimed to assess uptake of the studies reviewed; to contribute to analysis of the status of current MHPSS intervention research; to better understand knowledge transfer, use and impact; to analyse new dimensions for research; and to produce user-centred recommendations for research to better support humanitarian practice.

Based on the literature review and overall study objectives, the research team drafted a topic guide (Nemiro *et al*., 2021) as a platform for designing specific interview and survey tools for data collection. The tools were also informed by Elrha R2HC programme's four research impact areas: conceptual impact, instrumental impact, capacity and enduring connectivity (Tilley *et al*., 2018). The research tools were designed to be cross-cutting, include key profession-specific questions and tailored to the context of the research sites and the target groups being engaged. Key informant interviews followed a semi-structured interview guide. Questions were reviewed and refined during data collection in response to themes arising during the interviews. An online survey was created using Google Forms, with check boxes to record the majority of answers, although certain questions allowed for more detailed qualitative responses.

The sampling of interview participants was purposive and designed to reflect various professional, geographical and gender configurations that well represent this group of informants. The online survey was shared widely through platforms and resource hubs with MHPSS researchers and practitioners (e.g. through MHPSS.net and mhinnovation.net) with the aim of capturing a cross-section of these two participant groups. The final sample included 19 key informant interview participants, which took place between May and June 2020 (two key informants requested to be interviewed together), and 52 survey respondents (see Table 5 for full listing).

**Table 5.** Consultation data collection method, number of respondents

Method	Respondents	Number	Geographical region	Gender
Key informant interviews	MHPSS researchers (Re)	5	Europe-7	14 identified as female and five as male
	MHPSS practitioners (Pr)	5	Africa-5	
	Global MHPSS coordinators (Co)	4	Global-4	
	National government MHPSS focal persons (NaGov)	2	North America-2 Middle East-1	
	European governments/policymakers who fund MHPSS (DoG)	3		
	**Subtotal**	**19**		
Online survey	MHPSS researchers	20	Europe-19	33 identified as female, 16 as male, and three preferred not to say
	MHPSS practitioners	32	Africa-15	
	**Subtotal**	**52**	North America-5	
			Asia-7	
			Middle East-3	
			Latin America and the Caribbean-2	
			Oceania-1	
	**Total**	**71**		

Two researchers conducted the qualitative key informant interviews via Zoom, one leading the interview using the voice function (i.e. without video), and a second taking detailed notes of the interview content (also without video). Questions and answers were transcribed during the interview, cross-referenced with the sound recording to ensure clarity and annotated with comments and analysis.

The quantitative survey data were analysed using descriptive statistics on Excel software. Thematic analysis was used for the qualitative data. A coding scheme was developed based on the broad themes of the topic guide and from the initial hand coding of the interview data. This involved systematically sorting through the data, labelling ideas and phenomena as they appeared and reappeared. The researchers trialled the coding scheme to isolate discrepancies and revised the scheme accordingly. All interview data were coded and analysed, with the coded transcripts being reviewed by a second researcher. The trends that emerged were critically analysed according to the study's aims.

### Ethics approval

All procedures were approved by the Research Ethics Board of the London School of Economics (#2090). Informed consent was obtained from key informants and survey respondents, including the consent to use anonymised quotes in reporting and publications.

## Results

A total of 11062 references were generated from the searches. After excluding duplicates and screening from title and abstract, the full-text reports of 737 remaining citations were retained and screened. A total of 50 research studies were included in the final review, of which one contributed to two study reports from two different data pools (Betancourt *et al*., 2014; McBain *et al*., 2015). Of the studies included, 20 presented data on participant experiences/perspectives (including from process evaluations) using mixed-methods and qualitative data, and 30 were focused on outcomes, using primarily quantitative data. See Fig. 1 for the flow of studies through the review.

The majority of studies looking at participant experiences and perspectives (including process evaluations) (Barron and Abdullah, 2012; Walstrom *et al*., 2012; Richters *et al*., 2013; Eyber *et al*., 2014; Hogwood *et al*., 2014; Lilley *et al*., 2014; McBain *et al*., 2015; Schafer *et al*., 2015; Aldersey *et al*., 2016; Eiling *et al*., 2016; El-Khani *et al*., 2016; Hechanova *et al*., 2016; Hugelius *et al*., 2016; Asghar *et al*., 2018; Tol *et al*., 2018*b*; Greene *et al*., 2019; King, 2019; Koegler *et al*., 2019; Ordóñez-Carabaño *et al*., 2019; Sullivan *et al*., 2019) were scored as either ‘high’ or ‘medium’ for reliability and usefulness. Across the RCTs (Jordans *et al*., 2010; Betancourt *et al*., 2014; O'Callaghan *et al*., 2013; Lykes and Crosby, 2014; O'Callaghan *et al*., 2014; Aber *et al*., 2015; Blattman *et al*., 2015; Puffer *et al*., 2015; O'Callaghan *et al*., 2015; Puvimanasinghe and Price, 2016; Rahman *et al*., 2016; Hallman *et al*., 2018; Khan *et al*., 2017; Tol *et al*., 2018*b*; Sijbrandij *et al*., 2020; Tol *et al*., 2020*a*, 2020*b*), there was a predominance towards a low risk of bias for each of the domains scored, except for allocation concealment, blinding of participants and personnel, and allocation concealment, which were more ‘unclear’. There was a much higher risk of bias and a greater lack of clarity amongst the 12 controlled before-and-after studies (Ager *et al*., 2011; Sonderegger *et al*., 2011; Morris *et al*., 2012; Jordans *et al*., 2013; Mpande *et al*., 2013; Mercy Corps, 2015; Uyun and Witruk, 2017; Akiyama *et al*., 2018; Veronese and Barola, 2018; Metzler *et al*., 2019*a*, 2019*b*; Ziveri *et al*., 2019). Aside from risk of bias, six of the RCTs and controlled before-and-after designs (Jordans *et al*., 2013; Blattman *et al*., 2015; Puvimanasinghe and Price, 2016; Rahman *et al*., 2016; Veronese and Barola, 2018; Khan *et al*., 2017) had an additional qualitative component which increased their usefulness. Amongst the two cohort studies (Ager *et al*., 2010; McKay *et al*., 2011), one received an overall fair quality score, and another received a poor-quality score.

### Synthesised findings from the review and consultation

These findings are synthesised from the literature review and insights from the consultation process to answer two broad questions: (1) How has knowledge on MHPSS interventions researched in humanitarian settings advanced in the past 10 years? And (2) How has MHPSS intervention research been taken up in policy and practice at various levels of the humanitarian system? To answer these questions within the frame of the data collected, the results are structured around seven core themes that emerged from the literature and the consultation process. The core themes that emerged to answer question one include (1) an unbalanced evidence base, (2) a broadening in scope, but with mismatched outcome measures and (3) the influence of the RCT. The core themes that emerged to answer question two include (4) some progress in policy and practice, (5) a geographic divide between decision-making and locally perceived needs, (6) a disconnection of country-level MHPSS practitioners and (7) hindrances to uptake.

1. An unbalanced evidence base

There was a geographic emphasis in the literature towards East, West and Central Africa, where two-thirds of the studies reported were located (Ager *et al*., 2010, 2011; McKay *et al*., 2011; Sonderegger *et al*., 2011; Morris *et al*., 2012; Walstrom *et al*., 2012; Jordans *et al*., 2013; O'Callaghan *et al*., 2013, 2015; Richters *et al*., 2013; Betancourt *et al*., 2014; Eyber *et al*., 2014; Lykes and Crosby, 2014; Hogwood *et al*., 2014; Aber *et al*., 2015; Blattman *et al*., 2015; McBain *et al*., 2015; Puffer *et al*., 2015; Aldersey *et al*., 2016; Eiling *et al*., 2016; Hallman *et al*., 2018; Tol *et al*., 2018*a*, 2018*b*; Greene *et al*., 2019; King, 2019; Ordóñez-Carabaño *et al*., 2019; Koegler *et al*., 2019; Metzler *et al*., 2019*a*, 2019*b*; Sijbrandij *et al*., 2020; Tol *et al*., 2020*a*, 2020*b*). Despite considerable humanitarian work occurring in the Middle East and in Asia (the latter generally characterised by natural disasters), these settings had relatively limited representation in the literature.

The most commonly researched interventions were at the group level (Ager *et al*., 2010, 2011; Jordans *et al*., 2010; McKay *et al*., 2011; Sonderegger *et al*., 2011; Barron and Abdullah, 2012; Morris *et al*., 2012; Walstrom *et al*., 2012; Jordans *et al*., 2013; Mpande *et al*., 2013; O'Callaghan *et al*., 2013, 2014, 2015; Richters *et al*., 2013; Betancourt *et al*., 2014; Eyber *et al*., 2014; Hogwood *et al*., 2014; Lykes and Crosby, 2014; Lilley *et al*., 2014; Aber *et al*., 2015; Blattman *et al*., 2015; McBain *et al*., 2015; Puffer *et al*., 2015; Schafer *et al*., 2015; Mercy Corps, 2015; Aldersey *et al*., 2016; Eiling *et al*., 2016; Hechanova *et al*., 2016; Uyun and Witruk, 2017; Akiyama *et al*., 2018; Hallman *et al*., 2018; Asghar *et al*., 2018; Tol *et al*., 2018*a*, 2018*b*; Greene *et al*., 2019; King, 2019; Ordóñez-Carabaño *et al*., 2019; Koegler *et al*., 2019; Metzler *et al*., 2019*a*, 2019*b*; Sijbrandij *et al*., 2020; Tol *et al*., 2020*a*, 2020*b*) with individual (Blattman *et al*., 2015; Schafer *et al*., 2015; Puvimanasinghe and Price, 2016; Rahman *et al.*, 2016; Greene *et al*., 2019; Sullivan *et al*., 2019) and school-wide (Aber *et al*., 2015; Akiyama *et al*., 2018; Veronese and Barola, 2018) interventions the least researched. Interventions predominantly targeted adults, with limited direct evidence on outcomes for children and adolescents and no specific attention to whole family approaches. Where family-focused interventions were used, the interventions were primarily directed at parents and caregivers (e.g. parenting skills) and did not engage the whole family.

A small number of the studies reviewed integrated MHPSS within other technical areas; the most common were MHPSS-child protection (McKay *et al*., 2011; Eyber *et al*., 2014; O'Callaghan *et al*., 2015; Asghar *et al*., 2018; Mercy Corps, 2015; Hallman *et al*., 2018; Metzler *et al*., 2019*a*, 2019*b*), MHPSS-economic support (Ager *et al*., 2010; Lilley *et al*., 2014; Aldersey *et al*., 2016; Hallman *et al*., 2018; Koegler *et al*., 2019) and MHPSS-GBV (Gender-Based Violence) (O'Callaghan *et al*., 2013; Richters *et al.*, 2013; Hallman *et al*., 2018; Greene *et al*., 2019; Koegler *et al*., 2019). The nature of what counted as ‘integration’ varied across these studies and included those that incorporated programmatic components usually seen in another sector into their intervention design (e.g. the MHPSS-economic support studies integrated cash, economic support and group fund-shares into the interventions). Key informants also noted a lack of research on longer-term integrated MHPSS programming that addresses the most pressing and urgent humanitarian priorities (e.g. cash, shelter, food distribution and livelihoods).

Apart from a number of interventions for Child/Youth Friendly Spaces (Eyber *et al*., 2014; Mercy Corps, 2015; O'Callaghan *et al*., 2015; Metzler *et al*., 2019*a*, 2019*b*), there were minimal replications of the same approach in different settings in the literature reviewed. A relatively low proportion of the studies examined long-term impacts of the interventions with follow-up data collection or, if the follow-up did occur, this was not reflected in the literature. However, a significant proportion of interventions were adapted to the specific sociocultural context before being tested. Most of the studies reviewed used adapted assessment tools, actively adapted assessment tools or created new assessment tools to fit the setting and context, while a number of the studies described the development of locally relevant tools and incorporated these into their assessment processes.

Amongst the two research priorities developed by Tol *et al*. (2011*a*, 2011*b*) that were observable in the scope of this study (see Table 6), the literature review highlighted encouraging progress on research that adapts interventions to the sociocultural context (priority 5). However, the literature review and consultation process emphasised a continued lack of studies focusing on family-based interventions that measure family-oriented outcomes (priority 6) and school-based interventions to prevent, protect and promote psychosocial wellbeing and mental health among children and adults (priority 7). The body of MHPSS intervention research reviewed also did not consistently measure the appropriateness of its methods of assessing need (priority 8), nor did it show how interventions were explicitly responding to locally perceived needs (priority 10).

1. A broadening in scope, but with mismatched outcome measures

**Table 6.** Priority research questions (Tol *et al*., 2011*b*)

Research area	Research question
Problem analysis	1. What are the stressors faced by populations in humanitarian settings?
	2. How do affected populations themselves describe and perceive mental health and psychosocial problems in humanitarian settings?
	3. What are the major protective (including individual factors such as coping and hope) and contextual factors (e.g. justice mechanisms, religious practices) for mental health and psychosocial problems in humanitarian settings?
	4. Which are the most common mental health and psychosocial problems in the general population in humanitarian settings?
Mental health and psychosocial support interventions	5. How can we best adapt existing MHPSS interventions to different sociocultural contexts?^a^
	6. What is the effectiveness of family-based interventions to prevent mental disorders and protect and promote psychosocial wellbeing and mental health amongst children and adults living in humanitarian settings?
	7. What is the effectiveness of school-based psychosocial and mental health interventions to prevent mental disorders and to protect and promote psychosocial wellbeing and mental health among children and adults in humanitarian settings?
Research and information management	8. What are appropriate methods to assess mental health and psychosocial needs of populations in humanitarian settings?^a^
	9. What are appropriate indicators to use when monitoring and evaluating the results of mental health and psychosocial support in humanitarian settings?
Mental health and psychosocial support context	10. To what extent do current MHPSS interventions address locally perceived needs?

a Indicates the research priority was observable in the scope of this study.

The conclusions of key informants pointed to a broadening in scope and range of research over the last 10 years. Moving from a focus mostly on mental disorders and ‘dysfunction’ to using more positive outcome measures of mental health and psychosocial wellbeing that give greater attention to context. The literature review found that a total of 23 of the interventions studied were described as purely focused on promoting wellbeing/resilience (Ager *et al*., 2010, 2011, 2015; McKay *et al*., 2011; Eyber *et al*., 2014; Hogwood *et al*., 2014; Mercy Corps, 2015; O'Callaghan *et al*., 2015; Puffer *et al*., 2015; Schafer *et al*., 2015; Hechanova *et al*., 2016; McBain *et al*., 2015; Eiling *et al*., 2016; Akiyama *et al*., 2018; Asghar *et al*., 2018; Hallman *et al*., 2018; Veronese and Barola, 2018; King, 2019; Koegler *et al*., 2019; Ordóñez-Carabaño *et al*., 2019; Metzler *et al*., 2019*a*, 2019*b*; Sijbrandij *et al*., 2020), 18 were described as focusing on both promoting wellbeing/resilience and reducing suffering/dysfunction (Sonderegger *et al*., 2011; Barron and Abdullah, 2012; Morris *et al*., 2012; Jordans *et al*., 2013; Mpande *et al*., 2013; Richters *et al*., 2013; Lilley *et al*., 2014; O'Callaghan *et al*., 2014; Aldersey *et al*., 2016; El-Khani *et al*., 2016; Puvimanasinghe and Price, 2016; Hugelius *et al*., 2016; Khan *et al*., 2017; Uyun and Witruk, 2017; Tol *et al*., 2018*a*, 2018*b*; Greene *et al*., 2019; Ziveri *et al*., 2019), and nine were focused on only reducing suffering/dysfunction (Walstrom *et al*., 2012; O'Callaghan *et al*., 2013; Betancourt *et al*., 2014; Lykes and Crosby, 2014; Blattman *et al*., 2015; Rahman *et al*., 2016; Khan *et al*., 2017; Tol *et al*., 2018*a*; Sullivan *et al*., 2019). Key informants also noted an increased interest in research on the determinants of mental health and psychosocial wellbeing (e.g. poverty, GBV, marginalisation). This shift in research focus was felt to mirror the shift in practice at the global level and was expected to continue.

A key informant (Re 5) stated,

‘I anticipate a big shift…[to continue] over the next 10 years. The Lancet Commission on Global Mental Health was clear on that. Taking a full spectrum approach, moving away from the dichotomy of disorder versus non-disorder, but I think we are at the beginning of that. I think the research domain, if you aware of looking at MHPSS as a continuum, has been dominated by the ‘MH’ part…In fact, we are now putting a number of proposals together that are looking at issues of social connectedness, much more mechanistic outcomes, for example understanding the interplay between social determinants and mental health. That is part of the shift we are all in.’

However, a disconnect was identified in many of the studies reviewed, whereby the interventions were broad, community-based and geared towards positive outcomes, but the outcome measures used focused only on changes in symptoms of mental disorder. In several cases, there was a mismatch between the stated intervention aims and the focus of the research (including selected outcome measures). For example, in 12 studies (Eyber *et al*., 2014; Aber *et al*., 2015; Mercy Corps, 2015; O'Callaghan *et al*., 2015; Puffer *et al*., 2015; Schafer *et al*., 2015; Asghar *et al*., 2018; Hallman *et al*., 2018; Veronese and Barola, 2018; Koegler *et al*., 2019; Metzler *et al*., 2019*a*, 2019*b*), the intervention was described as purely focused on promoting wellbeing/resilience, and yet the research design included a focus on suffering/dysfunction. Key informants explained the previous focus on symptoms and disorders as related to the historical roots of MHPSS from medicine and psychiatry; the existing, validated measurement tools being mostly disorder-focused, which lend themselves better to RCTs; and the primary biomedical paradigm of certain high-impact academic journals.

1. The influence of the RCT

Having RCTs that demonstrate the effectiveness of MHPSS interventions was seen by key informants as advancing the field: ‘everyone loves an RCT, it adds an extra level of credibility to interventions’ (Pr 1). Indeed, amongst the new interventions that have been adopted into MHPSS practice (see below), many have been researched through RCTs, indicating the influence of these research designs. The limitations of this research design were also acknowledged by key informants, for example, being less readily applied to community-focused interventions that address difficult-to-measure changes and complex social dynamics. The literature review confirmed that person-focused psychological interventions have been disproportionately studied through RCTs, whereas community-focused interventions, including reintegration programmes, reconciliation and healing interventions, community-led support and community support groups, were less likely to have a controlled design. A risk associated with this was noted by one global coordinator:

‘RCTs are good for studying certain sorts of problems and interventions. This creates a bias around what studies are included in systematic reviews, which skews your view of what works.’ (Co 1)

Although key informants agreed that RCTs are important to demonstrate effectiveness, given the controlled conditions required for an RCT, many felt that other types of research design are required to understand how an intervention works in real-world programme implementation. MHPSS evidence generated more recently was acknowledged to include ‘a greater range of methods’, and key informants also concluded that the global MHPSS community was increasingly placing greater ‘legitimacy’ and value on qualitative research. The literature review was able to capture this broader range of designs, including controlled before-and-after studies, mixed-methods and qualitative designs.

1. Some progress in policy and practice

The consultation process indicated that MHPSS intervention research conducted over the last 10 years has influenced programmatic changes and uptake for several interventions, particularly scalable psychological interventions and a smaller number of broader, community-based interventions. Although not all intervention research has led to continued change or new programming, the following examples identified in the literature review and by key informants have been instrumental: (1) contextual refinement and implementation of newly developed scalable psychological interventions [e.g. Self Help Plus (Tol *et al*., 2018*a*), Problem Management Plus (WHO, 2018) and Interpersonal Psychotherapy-Group (WHO and Columbia University, 2016)], (2) further development of existing interventions [e.g. Advancing Adolescents (Panter-Brick *et al*., 2017) in Jordan and Syria], (3) influencing uptake and scaling of various interventions because of a supporting evidence base [e.g. informants reported that the fact that Psychological First Aid (PFA) is an ‘evidence-informed’ approach encouraged its wide uptake], and (4) re-evaluating and improving existing approaches [e.g. multi-country evaluation of child-friendly spaces (Hermosilla *et al*., 2019) led to a critical re-evaluation of the approach and greater attention to quality safeguards in its implementation].

Practitioner survey respondent reported that MHPSS intervention research has also influenced their choice of specific components of MHPSS approaches (e.g. the use of lay mental health workers, a focus on coping strategies and engagement with families), even when a component was not delivered in the context of the overall intervention in which it was originally studied. Key informant practitioner key informant confirmed that research had influenced their choice of overall intervention, although they often placed greater value on locally produced information, including the routine data they collected from needs assessments, lessons learned reports, and routine monitoring and evaluation activities to influence programming. The importance of *being seen* to be basing programmes on evidence was also raised, and one global MHPSS coordinator (headquarter-based) key informant noted*:* ‘there is an urgency and desire to say we are evidence-based’ (Co1). Yet, despite progress, programmes not being based on evidence were described as the reality in many contexts: ‘a lot of NGOs run MHPSS programming that has no evidence-base’ (Re1).

At the level of global MHPSS policy, research was thought by key informants to have influenced the shift from ‘critical incident stress debriefing’ to broad endorsement of PFA, and the shift from a single focus on trauma counselling and PTSD-focused interventions to transdiagnostic and community-based interventions. Global Child Protection policy has also shifted in response to a multi-country intervention study on child-friendly spaces. There was also a reported increased focus on and allocation of resources for adapting interventions based on rigorous needs assessments, consultations with end users and engagement with community-based stakeholders. However, amongst a body of 24 global MHPSS guidelines and strategy documents reviewed as part of the literature review process, only seven of the selected studies were cited once (Ager *et al*., 2010; Jordans *et al*., 2010; Morris *et al*., 2012; O'Callaghan *et al*., 2013; Puffer *et al*., 2015; Eiling *et al*., 2016) or twice (2). In addition, 11 studies (Ager *et al*., 2010, 2011; Jordans *et al*., 2010, 2013; O'Callaghan *et al*., 2013, 2014, 2015; Betancourt *et al*., 2014; Blattman *et al*., 2015; McBain *et al*., 2015; Eiling *et al*., 2016) were cited indirectly through their inclusion in systematic reviews which were referenced in the MHPSS guidelines and strategy documents.

Furthermore, only one example of an instrumental change in national government policy was reported by key informants, resulting from research conducted on the Advancing Adolescents programme in Jordan. The consultation process identified that other programme and/or policy change may have occurred without being documented or tracked as 45% (*n* = 9) of researchers surveyed noted that they do not systematically gather information on the changes that result from their research.

1. A geographic divide between decision-making and locally perceived needs

Academic researchers reported being under pressure from university requirements to secure publications in highly rated academic journals (‘that does change my focus quite a bit’, Re 1), and from funders who can act as ‘arbiters’ over the final topic of research. A geographic divide was identified in the hierarchy of ideas of the ‘global north’ over the ‘global south’ and supported by structural inequalities in research funding. One national government focal person described ‘ideas coming from the North and [being] sold to professionals in the South’ (NaGov 2). The survey asked researchers for the single most important reason behind their choice of current/most recent MHPSS intervention research topic out of five options and found that 40% (*n* = 8) reported that personal interest/continuation of previous research was the most important influence. Key informants recognised that MHPSS intervention research topics still need to be better ‘driven by needs in the field’ and not just ‘a good idea from a researcher behind the table’ (Co 2). A shift in research being informed by local need was partially observable from the literature reviewed; however, a theme emerged from the consultation process that MHPSS intervention research still remains top-down.

1. Disconnection of country-level MHPSS practitioners

During the interview process, the country-level based MHPSS practitioners described feeling ‘not very familiar’ (Pr3) with global research and research generated in country settings beyond their own, and none had heard of the 2010 MHPSS research priorities. This extended into a wider theme of ‘fragile knowledge’ and the disconnection of country-level based MHPSS practitioners from formal MHPSS intervention research findings. Country-level based MHPSS practitioners felt particularly disconnected from certain types of research, for example, research published primarily in academic journals: ‘Another thing – who has access to journals? Field-based practitioners don't have access’ (Co1). Although this lack of access to journals may be true for many country-based practitioners, headquarter-based key informants still reported that practice has been impacted by research, and this was further supported by survey findings.

The survey results demonstrated that 80% (*n* = 8) of practitioners surveyed who were based in Europe and North America rated their familiarity with MHPSS intervention research from the past 10 years as either four or five out of five, but only 36% (*n* = 2) of practitioners based outside of Europe and North America did so. When asked how easy it is to access MHPSS research, 60% (*n* = 6) of practitioners in Europe and North America reported a four or five out of five, compared to only 27% (*n* = 6) of those based outside Europe and North America. Ninety-four per cent (*n* = 30) of all practitioners agreed somewhat or very much that information is expensive or in difficult-to-access closed communities or portals. The findings indicated that the pathway that research travels through organisations and into practice is multi-layered involving several different actors at different levels, each likely engaged in their own preferred channels.

1. Hindrances to uptake

Country-level practitioners interviewed raised concerns about their ability to interpret and think critically about MHPSS intervention research. Not possessing these skills and knowledge was thought to risk under-appreciating the significance of research generated, misreading or over-stating the significance of research generated, and mis-applying findings and recommendations.

A further limit to the knowledge brokering process was that practitioners and policymakers lacked the time to access and become familiar with research. Ninety-four per cent of the practitioners surveyed agreed somewhat or very much that they lacked time to search for and engage in research learnings. As noted by one policymaker who was interviewed, this can present a ‘Catch-22’:

‘Maybe we are also at fault as policymakers in the sense that we don't read much. We are bogged down with issues of administration, planning, but with planning we need to plan with evidence.’ (NaGo2)

The step of actively transforming and translating evidence from knowledge to practice was raised across all the stakeholder groups interviewed as being critical to the knowledge brokering process. The majority suggested that evidence products should be digestible, practical and focused on the end user, without losing the nuance of the findings. The following quotes are representative of the different stakeholder groups engaged.

‘How research findings can be digested in a way that is easy to understand and see how it related… How evidence can lead to higher quality, more effective programming.’ (Co3)

‘Key challenges with research projects and findings is the communication of them and that often they are not targeted to who needs to receive them.’ (DoG1)

‘We need to simplify the information. One-page policy brief, giving infographics if there is a need for that.’ (NaGo2)

## Discussion

This study sought to answer how MHPSS interventions researched in humanitarian settings have advanced in the past 10 years and how the evidence has been adopted into policy and practice at various levels of the humanitarian system. The study was unique in that it focused on MHPSS interventions delivered in humanitarian settings that were *not* focused on mental disorders which allowed for a broader perspective more likely to capture interventions commonly implemented in MHPSS programming.

The literature review identified a rapidly growing evidence base that has evaluated a range of MHPSS interventions, and that gives greater attention to adapting interventions to the sociocultural context, which was one of the key research priorities identified by Tol *et al*. (2011*a*, 2011*b*) (priority 5, Table 6). However, there remains limited direct evidence on outcomes for children and adolescents and whole family approaches which were two further research priorities identified by Tol *et al*. (2011*a*, 2011*b*) (priority 6 and 7, Table 6). Few studies examined long-term impacts of interventions and there were minimal replications of the same approach that could test efficacy across settings and population groups. Key informants also noted a lack of research on longer-term integrated MHPSS programming that addresses the most pressing and urgent humanitarian priorities (e.g. cash, shelter, food distribution and livelihoods).

A general shift was identified in the consultation process away from a focus on disorder and towards the more positive aspects of wellbeing. However, there remained a mismatch in many studies included in the literature review, whereby the interventions were broad, community-based and geared towards positive outcomes, but the outcome measures used still focused on changes in symptoms of mental disorders. A number of the published studies excluded from the literature review for focusing on a clinical population reflected that they had delivered interventions with preventative or promotive objectives, but measured mental disorder symptoms and (perhaps unsurprisingly) found minimal or no change on these outcomes (e.g. Bass *et al*., 2012; Kohrt *et al*., 2015; Jani *et al*., 2016; Dhital *et al*., 2019). This mismatch has been observed in other reviews of MHPSS intervention research (Haroz *et al*., 2020; Purgato *et al*., 2018*a* ). Sometimes this may be appropriate, but more often it fails to capture the breadth of outcomes that could be generated by these types of interventions (e.g. social cohesion, social connectedness, functioning, agency) and thus restricts potential learning for programming and policy. These constructs may be more difficult to measure given the paucity of available validated scales, however progress is being made. For example, the IASC MHPSS Common Monitoring and Evaluation Framework, Version 2.0 (IASC, 2021) includes qualitative and quantitative means of verification for each impact indicator. Lessons can also be learned from research conducted in high-income countries, which have made more progress in this regard, and applied to research conducted in humanitarian settings.

Several newly implemented, scalable psychological interventions have been studied through RCTs suggesting the influence of research design on uptake. Conducting RCTs has built important credibility in the field, and this continues to be a high priority, but should not lead to exclusion of research on the types of interventions practitioners are actually implementing or want to implement. RCTs tend to focus more on person-centred interventions and individual mental health outcomes and exclude broad-based community interventions which are used more in practice (Bangpan *et al*., 2017; Bangpan *et al*., 2019). Such research can help to better understand and address the social determinants of mental health and psychosocial wellbeing such as poverty, interpersonal relationships, family dynamics and access to education (Bangpan *et al*., 2019). Moreover, integrated MHPSS programming that addresses the social determinants of mental health and psychosocial wellbeing is much harder to fit to an RCT mould. RCTs can also exclude more positive outcome measures of psychosocial wellbeing (Tol *et al*., 2011*a*, 2011*b*). A balance needs to be made between rigorous research and relevant research, or we risk increasing divisions between research and practice or implementing what is measurable through an RCT, rather than what is most appropriate to the context. Key stakeholders reported the need for other types of research designs, including those which can help to understand how an intervention works in complex humanitarian settings with the myriad challenges and instability that arises. Implementation research could be one way to achieve greater reach of research to practitioners, by exploring ways to understand the realities of day-to-day roll out, the cost-effectiveness of interventions and the various approaches to measuring and maintaining quality and fidelity.

MHPSS intervention research generated in the past 10 years has informed and influenced programming in humanitarian settings, especially around the implementation of newly developed scalable psychological interventions. Survey respondents reported that MHPSS intervention research also influenced their choice of specific components of MHPSS approaches, e.g. the use of lay workers, a focus on coping strategies, etc. Over the last few years, there has also been increased focus and allocation of resources for adapting evidence-based interventions with key community stakeholders. Instrumental change was reported at the level of global MHPSS policy; however, change was rare at the level of national government policy. Considering our finding that the impact of MHPSS intervention research has not been well tracked or documented, there may be other notable changes which have not been captured (e.g. the length of time it takes for research to result in notable programmatic changes). Supporting and tracking change should be the responsibility of all parties involved in generating and using evidence-based interventions; this especially pertains to the resources allocated by donors. Adequate time and funds are needed for uptake activities on research projects, and for monitoring and evaluation of these activities against specified outcomes, to ensure tangible change and demonstrable impacts for people and communities.

Researchers may find it challenging to isolate the changes which result from their research projects in emergency environments and monitoring the results of knowledge uptake is not a routine part of many research project or funding cycles. Supporting and tracking change should be the responsibility of all parties involved in generating and using evidence-based interventions; this especially pertains to the resources allocated by donors. Adequate time and funds are needed for uptake activities on research projects, and for monitoring and evaluation of these activities against specified outcomes, to ensure tangible change and demonstrable impacts for people and communities.

Despite the programme and policy changes reported, the study identified a general disconnect between country-level MHPSS practitioners and MHPSS intervention research. These findings have been highlighted previously (Tol *et al*., 2011*a*; Ventevogel, 2018). Decisions regarding MHPSS research topics appeared to remain more top-down than collaborative. The study noted a hierarchy of ideas that are supported by structural inequalities in research funding, and academic pressures placed on researchers. While this is not unique to the field of MHPSS and tends to be ubiquitous in humanitarian research (Leresche *et al*., 2020), it is imperative that effort is placed to ensure research is responsive to needs on the ground – in particular because this disconnection appears to be a major impediment to the uptake of research. Tol *et al*. (2020*a*, 2020*b*) highlight that the most studied MHPSS interventions are *still* not those that are widely utilised in humanitarian programming, and suggest that to minimise the disconnection, continued interactions amongst researchers and practitioners who are also decision makers are necessary.

Strong and inclusive researcher–practitioner collaboration can facilitate both higher quality and more relevant intervention research design, as well as support the interpretation and contextualisation of findings in ways that are most useful to the lived realities of practitioners and their programmes (Wright, 2020; UNICEF, 2021). From inception onwards, engaging with local practitioners on the development of research empowers them to be active participants for the entirety of the process, and this, in turn, supports advances in programming.

Useful collaborations can include a range of stakeholders such as researchers, global- and country-level MHPSS practitioners, local and national governments, and community stakeholders. Strong collaborations were seen by those engaged in this consultation process to result in: (1) mutual learning for all parties involved, (2) improved quality of research through the insights of partners, (3) buy-in for the intervention from key stakeholders and (4) more direct avenues for programme and policy change.

Greater investment in collaborative research by funders, researchers, practitioners and their organisations, policy makers and people with lived experience would build the capacity and competencies of those involved, increase the likelihood of sustained buy-in from key stakeholders, and generate direct avenues to influence programming and policy change.

## Limitations

The literature review was a scoping review rather than a systematic review, and in its balance of rigour and realism some relevant studies may not have been captured. By including only English language studies, relevant studies from non-Anglophone settings may have been omitted. Also, studies that focus on mental disorders were not included, but defining whether or not research is ‘disorder-focused’ is complex. Despite the robust rationale and rigorous selection process, another research team may have yielded different results. Using the search term ‘evaluation’ and ‘stress reduction’ *v*. (‘stress’ AND ‘reduction’) might have yielded different results.

Due to constraints from the COVID-19 pandemic, interviews were conducted remotely. As a number of potential key informants from national governments were engaged in their emergency response capacity, only two were interviewed. Fortunately, other key informants provided data on their interactions with national government actors, particularly senior practitioners and coordinators who work closely with this cohort. The same problem may have limited the number of respondents to the survey. The response to the survey was positive considering these circumstances; however, it was too small to disaggregate findings to a very granular level, e.g. comparing answers of community-based organisation staff with those working for international NGOs. The sample size was not large enough to provide statistically significant findings but was adequate for its intended purpose of triangulation with the qualitative interview data and considering the depth and detail of the form, the number of responses was judged to be satisfactory.
